# The Relationship Between Vitamin D Levels and Cardiac Remodelling in a Pediatric Dilated Cardiomyopathy Population: A Case-Control Study

**DOI:** 10.3390/jcdd12030082

**Published:** 2025-02-21

**Authors:** Asmaa Carla Hagau, Ioana-Octavia Matacuta-Bogdan, Lacramioara Eliza Chiperi, Beatrix-Jullia Hack, Iolanda Muntean

**Affiliations:** 1 Doctoral School of Medicine and Pharmacy, George Emil Palade University of Medicine, Pharmacy, Science, and Technology of Târgu Mureș, 540136 Târgu Mureș, Romania; 2 Clinic of Pediatric Cardiology, Emergency Institute for Cardiovascular Diseases and Transplantation of Târgu Mureș, 540139 Târgu Mureș, Romania; 3 Faculty of Medicine, “Lucian Blaga” University of Sibiu, 550025 Sibiu, Romania; 4 Department of Pediatrics, George Emil Palade University of Medicine, Pharmacy, Science, and Technology of Târgu Mureș, 540136 Târgu Mureș, Romania

**Keywords:** pediatric heart failure, vitamin D, dilated cardiomyopathy

## Abstract

Dilated cardiomyopathy (DCM) is a significant contributor to heart failure (HF) in the pediatric population despite its lower incidence compared to adults. Method: We present a case-control study that investigates serum levels of Vitamin D, measured as 25-hydroxyvitamin D (25-OHD), in children diagnosed with DCM and explores the relationship between Vitamin D levels and left ventricular (LV) dimensions and systolic function. Results: Thirty patients (mean age: 10.61 ± 6.54 years) with DCM were included, with a control group of thirty-one matched healthy children. We found a high prevalence of 25-OHD deficiency (67%) in the DCM group, which was statistically significant compared to the control group (*p* < 0.05). Notably, a significant negative correlation was observed between 25-OHD levels and both LV end-diastolic diameter (LVEDD; r = −0.43, *p* < 0.01) and end-systolic diameter (LVESD; r = −0.46, *p* < 0.01). However, no significant correlation was found between Vitamin D levels and LV ejection fraction or shortening fraction. Conclusion: These findings emphasise the importance of assessing Vitamin D status in pediatric DCM patients and may suggest that Vitamin D supplementation can be beneficial in managing this condition through its potential effects on cardiac remodelling and function. Further research is warranted to clarify the underlying mechanisms and therapeutic implications.

## 1. Introduction

Dilated cardiomyopathy (DCM) represents a significant problem within the pediatric population due to its critical role as a contributor to heart failure (HF) [1]. Unlike the adult population, where the incidence of DCM is 0.036–0.4%, the pediatric incidence is notably lower, between 0.003 and 0.006%. However, this condition poses severe long-term morbidity and mortality risks, particularly in infants under one year of age, where the prevalence rises to 8.34 cases per 100,000 inhabitants [2]. Additionally, epidemiological data indicate that male children are more frequently affected than females, with geographical variations also playing a role [3].

The pathophysiology of DCM reveals similar mechanisms in both adults and children, primarily involving decreased contractility of cardiomyocytes leading to left ventricular (LV) systolic dysfunction and HF. Also, the latest guideline from the European Society of Cardiology applies the same definition regarding cardiomyopathy to pediatric and adult pathology [1]. However, recent data suggest that there are some differences between pediatric and adult DCM pathophysiology regarding fibrotic and regenerative activity. First, distinct histological characteristics were seen, with fibrosis occurring mostly endocardially in children and interstitially in adults [4]. Second, pediatric patients often experience worse prognoses, indicated by myocardial cytolysis and hypertrophy [5,6]. Third, studies have shown that myocardial regenerative capacity in DCM is age-dependent, highest during early infancy, with a progressive decline with age [7]. Additionally, pediatric DCM shows distinct gene expression profiles, a higher potential for spontaneous cardiac recovery, and less reliance on disease duration, while adult DCM progression is influenced by factors like hypertension, chronic kidney disease, and disease duration, with limited recovery potential [8]. Also, a recent study identified distinct biomarker profiles in pediatric DCM versus adult DCM, highlighting the concept of age-specific pathophysiological mechanisms despite sharing similar clinical presentations [9].

Thus, recognising the distinct pathophysiological features involved between these two different populations is essential for appropriate management. Overall, pediatric DCM presents unique challenges and differences compared to its adult counterpart, highlighting the need for age-specific diagnostic and therapeutic approaches.

Although multiple risk factors for cardiovascular diseases are known, recent studies have focused on additional risk factors that may predict patient prognosis, such as Vitamin D deficiency. Vitamin D, a fat-soluble steroid hormone with multiple sources, is obtained either through diet or synthesised in the body [10]. The active metabolite of Vitamin D mediates its biological effects through different mechanisms. Directly, its synthesis occurs in various cell types because of the presence of the activating enzyme in multiple tissues. Indirectly, it exerts its effects by binding to vitamin D receptors (VDR), which are widely distributed across different cell types, highlighting their extensive physiological significance [11]. Emerging studies have highlighted a significant association between Vitamin D deficiency and HF. The reduced sun exposure from exercise intolerance and age-related inefficiencies in vitamin D synthesis propose an explanation for the high prevalence of Vitamin D deficiency in HF patients. However, increasing evidence links lower Vitamin D levels to greater disease severity, elevated natriuretic peptides, reduced exercise capacity, poorer quality of life, and an increased risk of mortality [12]. Consequently, researchers have proposed several hypotheses to explain this association.

First, Vitamin D plays a crucial physiological role in regulating cardiac function, particularly through its influence on calcium homeostasis in cardiomyocytes. Myocyte contraction depends on the coordinated interaction of calcium ions, which exert a positive inotropic effect on myocardial contractility and contractile proteins. At the same time, disruptions in calcium homeostasis are strongly associated with impaired contractility and cardiac dysfunction [12,13]. Second, several studies showed that loss of Vitamin D receptors causes dysregulation of the renin–angiotensin–aldosterone system, significantly elevating the levels of renin, angiotensin II, aldosterone and blood pressure. Notably, the alteration in the renin-angiotensin-aldosterone system has been associated with cardiac hypertrophy, as well as direct involvement in inflammatory and fibrotic processes, further contributing to the progression of HF [14,15].

It is important to mention that existing research primarily focuses on adults. [16,17]. However, the role of Vitamin D in pediatric cardiac pathology has not been extensively studied. Thus, the objective of this study is to evaluate the serum levels of Vitamin D by measuring the level of its metabolite 25-dihydroxy-Vitamin D (25-OHD) in children with DCM and investigate the correlation between their serum levels and the dimensions and systolic function of the LV in these patients.

## 2. Materials and Methods

The present observational case-control study was conducted at the Pediatric Cardiology Clinic, part of the Institute of Cardiovascular Diseases and Transplantation in Târgu Mureș, Romania, from January 2022 to December 2023. The study protocol was explained to all legal guardians or parents of the participants. Written informed consent was obtained from the parents or guardians of the participating children, both for their involvement in the study and for the publication of the resulting data. The study was conducted per the Declaration of Helsinki and approved by the Ethics Committee of the Emergency Institute of Cardiovascular Diseases and Transplant from Targu Mures.

### 2.1. Population Study

The study enrolled 30 patients diagnosed with DCM who were monitored at the Pediatric Cardiology Clinic, part of the Institute mentioned above (DCM Group). As a case-control study, the patients’ data were compared with a group of 31 matched healthy patients in terms of race, age and anthropometric characteristics (Control Group).

We included pediatric patients aged between 1 month and 18 years diagnosed with idiopathic DCM. Dilated cardiomyopathy was defined by the presence of an LV ejection fraction (LVEF) of ≤ 50% and left ventricular end-diastolic diameter (LVEDD) with a Z score of ≥2 SD.

The main exclusion criteria were patients over 18 years of age, patients diagnosed with secondary DCM due to hypertension, congenital heart diseases, coronary artery anomalies or valvular diseases, neurodegenerative diseases, patients with associated liver or kidney failure, and patients who received treatment with Vitamin D or any drug that interferes with Vitamin D.

### 2.2. Working Method

#### 2.2.1. Clinical and Biochemical Data

During the evaluations, the patient’s clinical data (classification of HF according to the NYHA/Ross class, anthropometric data) were recorded. The New York Heart Association (NYHA) and Ross classification are crucial for assessing heart failure severity in these patients and guiding management strategies [18].

Blood samples were taken from patients to determine the 25-OHD levels and analysed using the High-Performance Liquid Chromatography method. The level of 25-OHD was classified as deficient if the serum level was below 20 ng/mL, insufficient if it was between 20 and 30 ng/mL, and sufficient if it was above 30 ng/mL.

#### 2.2.2. Echocardiographic Protocol

Patients were evaluated echocardiographically using the Phillips Epiq echocardiograph, utilising 5–12 mHz probes. Echocardiographic parameters were assessed using the standard views: apical four-chamber and parasternal short and long-axis views of the LV. For evaluating LV systolic function, the LVEF and LV fractional shortening (LVFS) were obtained from the parasternal long-axis view using a two-dimensional guided M-mode echocardiography method. Also, LV dimensions (LVEDD and left ventricular end-systolic diameter (LVESD)) were obtained from the parasternal long-axis view using M-mode [19]. The z-scores were calculated using Cantinotti z-scores [20].

### 2.3. Statistical Tests Used

Statistical analysis was performed using GraphPad Prism 9, IBM SPSS 13.0, and Microsoft Excel, considering *p* < 0.05 statistically significant.

Normality tests were applied to all variables. The numerical description of the data was performed using the mean ± SD for parametric distributions and the median ± interquartile range (IQR) for non-parametric distributions. At the same time, categorical variables were presented as numbers/percentages. To compare the data between the two groups, parametric tests (unpaired *t*-test) or non-parametric tests (Mann–Whitney test) were used, as appropriate.

The correlation between variables was analysed using the Spearman–Pearson correlation test, with a *p*-value < 0.05 considered statistically significant.

## 3. Results

### 3.1. Main Characteristics of the Patients

As we previously stated, 30 patients diagnosed with DCM were included in the study. In the DCM group, 23% (7 patients) were included in NYHA/Ross class I, 30% (9 patients) in NYHA/Ross class II, 26% (8 patients) in NYHA/Ross class III and 20% (6 patients) in NYHA/Ross class IV. The main characteristics of the patients are shown in Table 1. Out of the 30 patients, 20 were male and 10 were female, aged between 3 months and 17 years. The mean age of patients included in the study was 14 (IQR 3–16) years for the DCM group and 15.3 (IQR 9.8–16.6) years for the control group. The male-female sex ratio in both studied groups was 2:1. There were no statistical differences in age, sex or anthropometric characteristics between the studied groups.

### 3.2. Comparison Between Groups

**Figure 1:**
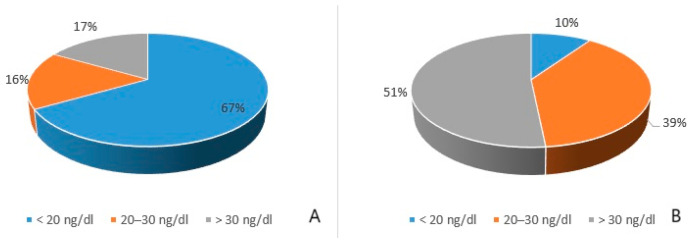
(**A**) Distribution of 25-OHD measurements in DCM group; (**B**) Distribution of 25-OHD measurements in control group.

**Figure 2:**
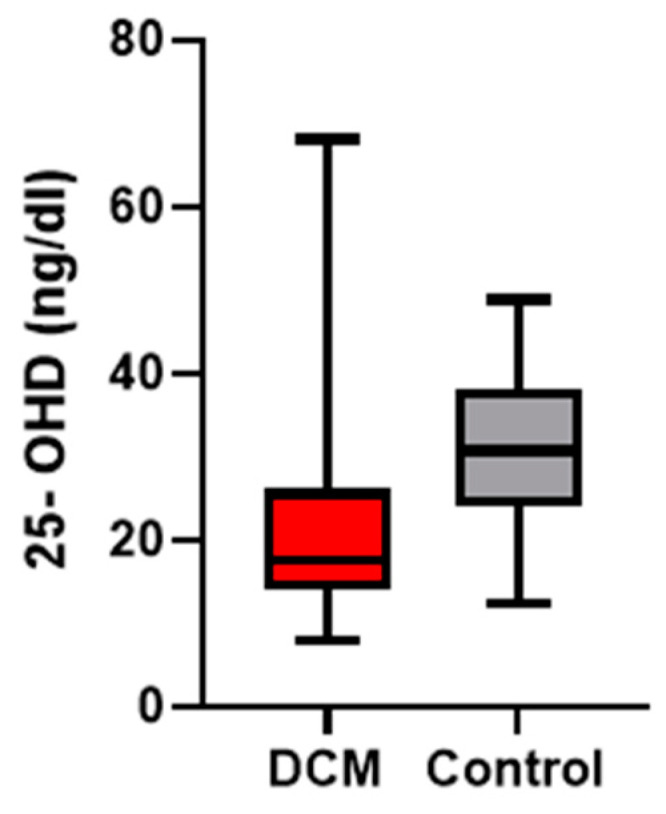
The box-and-whisker plot compares the serum levels of 25-OHD between the DCM and control groups. Median 25-OHD levels are visibly lower in the DCM group compared to the control group.

In the DCM group, the measurement of 25-OHD levels revealed that the majority of patients (67%) exhibited a deficiency of 25-OHD, 16% had insufficient levels, and 17% showed normal values. In the control group, 51% of patients exhibited normal 25-OHD levels (Figure 1), with statistically significant differences between the two groups (*p* < 0.05) (Figure 2).

As previously stated, all patients underwent echocardiographic evaluation to assess cardiac structure and function, as summarised in Table 2.

In the DCM group, the mean LVEF was 38.5 ± 16.4, indicating significant systolic dysfunction. Similarly, the LVFS was markedly reduced, with a mean of 18.71 ± 8.99%. Structural abnormalities were evident, with the LVEDD averaging 5.60 ± 1.37 cm, with a median Z-score of 3.33 (range 2.52–7.94). The LVESD showed a mean of 4.51 ± 1.45 with a median Z-score of 4.6 (range 2.85–14), suggesting advanced ventricular dilation and remodelling.

### 3.3. Results in the DCM Group

**Figure 3:**
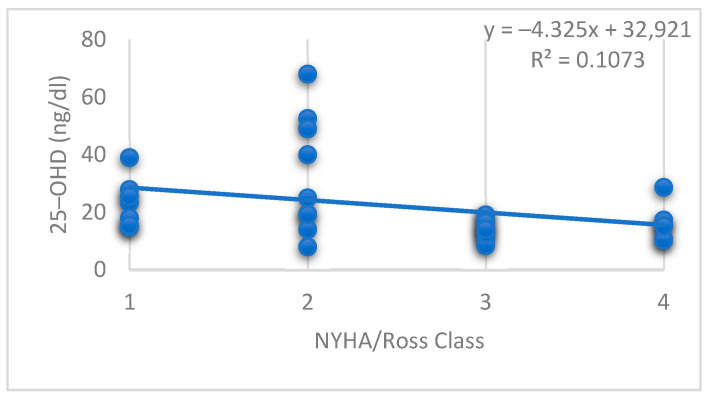
Correlation between 25-OH Vitamin D levels and NYHA/Ross Class.

Using the Spearman statistical test to analyse the relationship between 25-OHD levels and NYHA/Ross Class in the DCM group, the individual correlation coefficient r = −0.47 (*p* < 0.05) indicates a significant negative correlation between 25-OH Vitamin D levels and NYHA/Ross Class (Figure 3).

**Figure 4:**
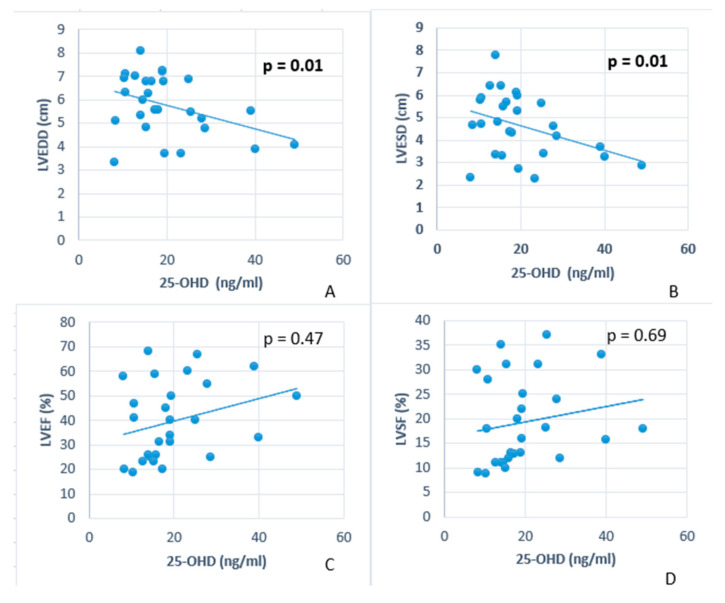
Correlation analysis revealed a significant inverse relationship between 25-OHD levels and LVEDD (**A**) as well as LVESD (**B**) in the DCM group. In contrast, no significant correlation was observed between 25-OHD levels and LVEF (**C**) or LVFS (**D**).

Using the Spearman statistical test to evaluate the relationship between 25-OHD levels and LVEDD in the DCM group, the correlation coefficient (r = −0.42, *p* < 0.05) reveals a significant negative correlation between these variables. Additionally, the analysis of 25-OHD levels and LVESD in the same group demonstrates a similar significant negative correlation (r = −0.48, *p* < 0.05) (Table 3, Figure 4).

However, when we analysed the relationship between 25-OHD levels and LVEF, the correlation coefficient (r = 0.138, *p* > 0.05) indicated no significant correlation between these variables (Table 3, Figure 4). A multiple linear regression analysis assessed whether 25-OHD levels, age, and BMI predict LVEF. The model did not identify any significant predictors of LVEF, as 25-OHD (β = −0.054, *p* = 0.833), age (β = 0.054, *p* = 0.861), and BMI (β = −0.150, *p* = 0.559) were all statistically non-significant. These findings suggest that 25-OHD levels, age, or BMI do not significantly influence LVEF. Also, non-linear regression analyses were performed to investigate the potential non-linear relationship between LVEF and 25-OHD levels. A quadratic model suggested a weak U-shaped association (R^2^ = 0.095), with LVEF initially increasing with 25-OHD levels. However, this relationship did not reach statistical significance (*p* = 0.273). The cubic model did not improve model fit (R^2^ = 0.095, *p* = 0.466). Overall, the results indicate that Vitamin D does not significantly predict EF, regardless of whether a linear or nonlinear model is applied.

Similarly, the correlation between 25-OHD levels and LVFS showed no significant relationship (r = 0.132, *p* > 0.05 (Table 3, Figure 4). Multiple regression models were applied to investigate the potential relationship between LVSF and 25-OHD levels. The analysis aimed to determine whether non-linear models could better capture the association between these variables compared to a standard linear approach. The linear regression model revealed a weak and non-significant association between SF and Vitamin D levels (R^2^ = 0.012, *p* = 0.573). Similarly, the logarithmic model (R^2^ = 0.001, *p* = 0.862) failed to demonstrate any meaningful relationship. Higher-order polynomial models were explored to assess potential non-linear effects. Still, neither model reached statistical significance, and the higher-order terms (quadratic and cubic coefficients) remained non-significant (*p* > 0.05). These results suggest that a linear relationship between SF and Vitamin D is unlikely in this dataset. None of the tested models provided significant evidence supporting a relationship between SF and Vitamin D levels. These findings indicate that Vitamin D is not a strong independent predictor of SF, regardless of the model specification.

## 4. Discussion

Dilated cardiomyopathy is a significant contributor to morbidity and mortality in the paediatric population, leading to severe HF. Vitamin D status has started to be increasingly studied in recent years among adult patients with HF due to studies in the literature that are beginning to demonstrate a significant correlation between vitamin D deficiency and HF. In the paediatric population, however, studies are still limited. In paediatric patients, vitamin D deficiency in those with HF and congenital heart disease has been associated with increased inotropic support requirements, a higher risk of prolonged hospitalisation, and postoperative infections [21].

Although the active metabolite of vitamin D is 1,25-dihydroxyvitamin D (calcitriol), its serum level is not considered an ideal indicator of vitamin D status because it is influenced by various external factors (serum phosphorus, parathyroid hormone, renal function) and has a short half-life [22]. Therefore, the pre-hormone 25-OH vitamin D (25-hydroxycholecalciferol) is considered a more reliable indicator for assessing vitamin D status due to its higher plasma concentration and longer half-life and is used in most studies [22]. Based on this, in our study, we measured 25-OHD to determine the vitamin D status of the studied patients.

Our study revealed a high prevalence of 25-OHD deficiency among paediatric patients with DCM, with only 17% of patients having sufficient 25-OHD levels. Additionally, these patients demonstrated significantly lower 25-OHD levels compared to healthy children. The results are aligned with the results in the adult population. For example, in a study conducted on 56 adults with DCM and HF, Priya et al. observed that serum levels of 25-OHD were significantly lower in patients with DCM and HF compared to a control group [23].

Also, we revealed a significant negative correlation between 25-OHD levels and the NYHA/Ross class of the patients with DCM. This indicates that lower levels of 25-OHD are associated with a higher NYHA/Ross classification, which is typically indicative of worse clinical status in HF patients. In the paediatric population, the evidence linking 25-OHD levels with clinical staging, such as NYHA or Ross Class, remains more limited and less robust compared to the adult population, largely due to differences in disease pathophysiology, treatment regimens, and age-related physiological factors. In a double-blind, placebo-controlled intervention study published by Shedeed et al. that included 80 infants with chronic HF, reduced HF Class was observed after 12 weeks of Vitamin D supplementation in the Vitamin D group, suggesting the beneficial effects of vitamin D supplementation [24]. In adult patients with HF, the relationship between vitamin D levels and clinical severity has been more extensively studied. For example, many studies have found that lower 25-OHD levels correlate with higher NYHA classifications and worse clinical outcomes, including increased mortality [25,26].

Additionally, our study revealed a negative correlation between vitamin D levels and LVEDD and LVESD in children with DCM. Similar findings were reported by Ameri et al., who found a negative correlation between vitamin D levels, LVESD, and left ventricular volume in patients with HF [27]. Also, in a study published by Pandit et al., vitamin D deficiency was significantly correlated with interventricular septum thickness and left ventricular mass index [28]. Furthermore, vitamin D deficiency was correlated with changes in LV geometry even in adults without HF [29]. These echocardiographic findings suggest that low vitamin D levels are associated with increased LV chamber dimensions, underscoring the profound cardiac dysfunction and structural remodelling characteristic of DCM in this population, reflecting the severity of the disease and its impact on myocardial performance.

Surprisingly, in our study, we did not observe a significant correlation between vitamin D levels and LVEF or LVSF. Interestingly, in a study analysing vitamin D levels in 44 paediatric patients with idiopathic DCM by Raafat et al., in addition to the negative correlations between vitamin D levels and LV diameters, a significant correlation was also found with LVFS, but with a statistical *p* of 0.047 [30]. However, more significant correlations were found in adult patients with DCM [31].

There are some possible explanations regarding these inconsistencies. First, LVEF and LVSF are echocardiographical measurements that primarily assess the contractile function with a load-dependent nature. Therefore, our findings may suggest that vitamin D deficiency can promote ventricular dilatation and remodelling without the immediate decline in the contractile function. It is known that cardiac remodelling in HF is a complex process that, regardless of the underlying pathology, follows a similar pathway that combines, at first, adaptive changes such as known compensatory mechanisms (increased preload and the Frank-Starling mechanism) that may preserve the systolic function in the early and compensated stages of LV remodelling [32]. Second, advanced myocardial echocardiography, such as speckle tracking, can provide more insights regarding the relationship between vitamin D deficiency and contractile function in paediatric patients. Furthermore, the stronger correlation between vitamin D deficiency and reduced LVEF in adult patients versus paediatric patients may be explained by the differences in cardiac adaptation, associated comorbidities, and also the duration of vitamin D deficiency exposure in adults. Further studies with a larger paediatric population sample size and advanced myocardial imaging may provide additional insights regarding the vitamin D relationship with LV contractility measurements.

Despite these inconsistencies, the role of vitamin D deficiency in altering the cardiac structure and function highlights its potential involvement in cardiac remodelling, suggesting that vitamin D deficiency can play a contributing role in the pathogenesis of DCM. Interestingly, isolated reports have documented paediatric DCM cases linked to hypocalcaemia and vitamin D deficiency, whose symptoms completely resolved after supplementation with calcium and vitamin D, emphasising the importance of considering vitamin D deficiency as a potentially reversible contributor to DCM in regions where nutritional rickets is endemic [33,34].

Due to the design of the study, several limitations must be noted. First, due to the observational design, even though we identified significant correlations between vitamin D deficiency and cardiac remodelling in a subgroup of paediatric HF patients, we could not prove that vitamin D deficiency is a direct factor in cardiac remodelling. Second, the small number of patients in the study group diagnosed in a single paediatric centre could impact the representativeness of the findings and limit generalisability to other paediatric populations. While our study involved a small group of patients, it is noteworthy that these individuals were evaluated at the largest paediatric cardiology centre in Romania, which serves as the primary referral institution for all patients with DCM and severe HF, given that it is the only paediatric heart transplant centre in the country. This context adds significance to our findings, considering the low incidence of the disease overall, and paediatric studies regarding DCM and vitamin D are still limited; therefore, this study can provide some insights for future research, such as randomised controlled trials or longitudinal trials for a better understanding of the casualty between vitamin D deficiency and paediatric HF. Third, the assessment of a single time point of vitamin D levels may not accurately reflect long-term vitamin D status. However, our findings emphasise the need to monitor vitamin D levels in children at risk for or diagnosed with DCM and HF and to identify vitamin D deficiency as a modifiable factor in this type of population that is vulnerable to deficiency.

## 5. Conclusions

In conclusion, emerging evidence suggests that vitamin D deficiency, known to influence myocardial function and remodelling, may exacerbate the pathogenesis of DCM in children, underscoring the need for comprehensive metabolic and nutritional evaluations in this population, early identification of risk factors and timely interventions, including optimised pharmacological therapies, is crucial for improving outcomes. Although additional studies are needed to elucidate the effects of Vitamin D on the myocardium, we think that Vitamin D supplementation in patients with vitamin D deficiency and HF could play an adjunctive role in treating heart failure and cardiac remodelling. Therefore, an integrated approach, including the evaluation of vitamin D status, could have a significant impact on the management of this condition.

## Figures and Tables

**Table 1:** Main characteristics of patients in the DCM group versus the control group.

	DCM Group (N = 30 Patients)	Control Group (N = 31 Patients)	*p*-Value
Sex	Male 20 patients (66%)	Male 19 patients (61,20%)	0.79
	Female 10 patients (33%)	Female 12 patients (38,71%)	
Age (yr)	10.61 ± 6.54	13.22 ± 4.48	0.18
0−5 years	No. 8 (26%)	No. 4 (13%)	
6−14 years	No. 7 (23%)	No. 9 (29%)	
14−17 years	15 (50%)	No. 18 (58%)	
Weight (kg)	41.4 ± 25.54	51.39 ± 16.45	0.16
Height (cm)	139 ± 42.56	157.5± 23.09	0.34
BMI (kg/m^2^)	18.25 ± 4.51	20.3 ± 2.802	0.06
BSA (m^2^)	1.23 ± 0.58	1.49 ± 0.35	0.24

yr—years; kg—kilograms; cm—centimetres; BMI—body mass index; BSA—body surface area (calculated with Montpeler); Data is presented as mean ± SD.

**Table 2:** Echocardiographical characteristics of the DCM group vs the control group.

	DCM Group		Control Group		*p* Value
Parameter	Mean ± SD	Z-Score	Mean ± SD	Z-Score	
LVEF (%)	38.5 ± 16.4		70.69 ± 4.98		<0.0001
LVSF (%)	18.71 ± 8.99		39.77 ± 4.39		<0.0001
LVEDD (cm)	5.60 ± 1.37	3.33 (2.5–7.94)	4.2 ± 0.67	<2.5	<0.0001
LVESD (cm)	4.51 ± 1.45	4.6 (2.85–14)	2.49 ± 0.43	<2.5	<0.0001

LVEF—left ventricle ejection fraction; LVSF—left ventricle shortening fraction; LVEDD—left ventricle end-diastolic dimension, LVESD—left ventricle end-systolic dimension; SD—standard deviation; cm—centimetres.

**Table 3:** Non-parametric Spearman correlations within the study group.

Variable	r Value	25-OHD—*p*-Value
LVEDD (cm)	−0.4297	0.01
LVESD (cm)	−0.455	0.01
LVEF (%)	0.138	0.47
LVSF (%)	0.132	0.69

## Data Availability

The data presented in this study are available on request from the corresponding author.
